# Planning and Conducting Clinical Research: The Whole Process

**DOI:** 10.7759/cureus.4112

**Published:** 2019-02-20

**Authors:** Boon-How Chew

**Affiliations:** 1 Family Medicine, Universiti Putra Malaysia, Serdang, MYS

**Keywords:** clinical epidemiology, literature review, conceptual framework, research question, study designs, study reporting

## Abstract

The goal of this review was to present the essential steps in the entire process of clinical research. Research should begin with an educated idea arising from a clinical practice issue. A research topic rooted in a clinical problem provides the motivation for the completion of the research and relevancy for affecting medical practice changes and improvements. The research idea is further informed through a systematic literature review, clarified into a conceptual framework, and defined into an answerable research question. Engagement with clinical experts, experienced researchers, relevant stakeholders of the research topic, and even patients can enhance the research question’s relevance, feasibility, and efficiency. Clinical research can be completed in two major steps: study designing and study reporting. Three study designs should be planned in sequence and iterated until properly refined: theoretical design, data collection design, and statistical analysis design. The design of data collection could be further categorized into three facets: experimental or non-experimental, sampling or census, and time features of the variables to be studied. The ultimate aims of research reporting are to present findings succinctly and timely. Concise, explicit, and complete reporting are the guiding principles in clinical studies reporting.

## Introduction and background

Medical and clinical research can be classified in many different ways. Probably, most people are familiar with basic (laboratory) research, clinical research, healthcare (services) research, health systems (policy) research, and educational research. Clinical research in this review refers to scientific research related to clinical practices. There are many ways a clinical research's findings can become invalid or less impactful including ignorance of previous similar studies, a paucity of similar studies, poor study design and implementation, low test agent efficacy, no predetermined statistical analysis, insufficient reporting, bias, and conflicts of interest [1-4]. Scientific, ethical, and moral decadence among researchers can be due to incognizant criteria in academic promotion and remuneration and too many forced studies by amateurs and students for the sake of research without adequate training or guidance [2,5-6]. This article will review the proper methods to conduct medical research from the planning stage to submission for publication (Table 1).

**Table 1:** Overview of the essential concepts of the whole clinical research process ^a^Feasibility and efficiency are considered during the refinement of the research question and adhered to during data collection.

Concept	Research Idea	Research Question	Acquiring Data	Analysis	Publication	Practice
Actions	Relevant clinical problem or issue	Primary or secondary	Measuring	Prespecified	Writing skills	Guidelines
	Literature review	Quantitative or qualitative	Measuring tool	Predetermined	Guidelines	Protocol
	Conceptual framework	Causal or non-causal	Measurement	Exploratory allowed	Journal selection	Policy
	Collaboration with experts	Feasibility^a^	Feasibility^a^	Strength and direction of the effect estimate	Response to reviewers’ comments	Change
	Seek target population’s opinions on the research topic	Efficiency^a^	Efficiency^a^			
		Theoretical Design	Data Collection Design	Statistical design		
		Domain (external validity)	Experimental or non-experimental	Data cleaning		
		Valid (confounding minimized)	Sampling or census	Outlier		
		Precise (good sample size)	Time features	Missing data		
		Pilot study		Descriptive		
				Inferential		
				Statistical assumptions		
				Collaboration with statistician		

Epidemiologic studies in clinical and medical fields focus on the effect of a determinant on an outcome [7]. Measurement errors that happen systematically give rise to biases leading to invalid study results, whereas random measurement errors will cause imprecise reporting of effects. Precision can usually be increased with an increased sample size provided biases are avoided or trivialized. Otherwise, the increased precision will aggravate the biases. Because epidemiologic, clinical research focuses on measurement, measurement errors are addressed throughout the research process. Obtaining the most accurate estimate of a treatment effect constitutes the whole business of epidemiologic research in clinical practice. This is greatly facilitated by clinical expertise and current scientific knowledge of the research topic. Current scientific knowledge is acquired through literature reviews or in collaboration with an expert clinician. Collaboration and consultation with an expert clinician should also include input from the target population to confirm the relevance of the research question. The novelty of a research topic is less important than the clinical applicability of the topic. Researchers need to acquire appropriate writing and reporting skills from the beginning of their careers, and these skills should improve with persistent use and regular reviewing of published journal articles. A published clinical research study stands on solid scientific ground to inform clinical practice given the article has passed through proper peer-reviews, revision, and content improvement.

## Review

Systematic literature reviews

Systematic literature reviews of published papers will inform authors of the existing clinical evidence on a research topic. This is an important step to reduce wasted efforts and evaluate the planned study [8]. Conducting a systematic literature review is a well-known important step before embarking on a new study [9]. A rigorously performed and cautiously interpreted systematic review that includes in-process trials can inform researchers of several factors [10]. Reviewing the literature will inform the choice of recruitment methods, outcome measures, questionnaires, intervention details, and statistical strategies – useful information to increase the study’s relevance, value, and power. A good review of previous studies will also provide evidence of the effects of an intervention that may or may not be worthwhile; this would suggest either no further studies are warranted or that further study of the intervention is needed. A review can also inform whether a larger and better study is preferable to an additional small study. Reviews of previously published work may yield few studies or low-quality evidence from small or poorly designed studies on certain intervention or observation; this may encourage or discourage further research or prompt consideration of a first clinical trial.

Conceptual framework

The result of a literature review should include identifying a working conceptual framework to clarify the nature of the research problem, questions, and designs, and even guide the latter discussion of the findings and development of possible solutions. Conceptual frameworks represent ways of thinking about a problem or how complex things work the way they do [11]. Different frameworks will emphasize different variables and outcomes, and their inter-relatedness. Each framework highlights or emphasizes different aspects of a problem or research question. Often, any single conceptual framework presents only a partial view of reality [11]. Furthermore, each framework magnifies certain elements of the problem. Therefore, a thorough literature search is warranted for authors to avoid repeating the same research endeavors or mistakes. It may also help them find relevant conceptual frameworks including those that are outside one’s specialty or system. 

Conceptual frameworks can come from theories with well-organized principles and propositions that have been confirmed by observations or experiments. Conceptual frameworks can also come from models derived from theories, observations or sets of concepts or even evidence-based best practices derived from past studies [11].

Researchers convey their assumptions of the associations of the variables explicitly in the conceptual framework to connect the research to the literature. After selecting a single conceptual framework or a combination of a few frameworks, a clinical study can be completed in two fundamental steps: study design and study report. Three study designs should be planned in sequence and iterated until satisfaction: the theoretical design, data collection design, and statistical analysis design [7]. 

Study designs

Theoretical Design

**Figure 1:**
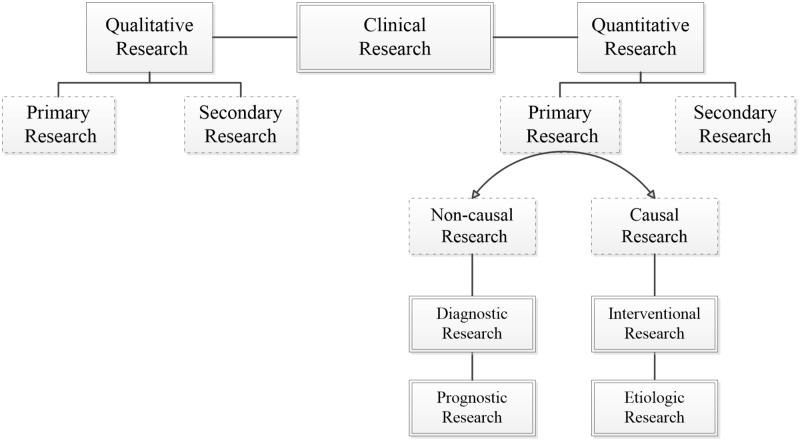
Fundamental classification of clinical studies

Theoretical design is the next important step in the research process after a literature review and conceptual framework identification. While the theoretical design is a crucial step in research planning, it is often dealt with lightly because of the more alluring second step (data collection design). In the theoretical design phase, a research question is designed to address a clinical problem, which involves an informed understanding based on the literature review and effective collaboration with the right experts and clinicians. A well-developed research question will have an initial hypothesis of the possible relationship between the explanatory variable/exposure and the outcome. This will inform the nature of the study design, be it qualitative or quantitative, primary or secondary, and non-causal or causal (Figure 1).

A study is qualitative if the research question aims to explore, understand, describe, discover or generate reasons underlying certain phenomena. Qualitative studies usually focus on a process to determine how and why things happen [12]. Quantitative studies use deductive reasoning, and numerical statistical quantification of the association between groups on data often gathered during experiments [13]. A primary clinical study is an original study gathering a new set of patient-level data. Secondary research draws on the existing available data and pooling them into a larger database to generate a wider perspective or a more powerful conclusion. Non-causal or descriptive research aims to identify the determinants or associated factors for the outcome or health condition, without regard for causal relationships. Causal research is an exploration of the determinants of an outcome while mitigating confounding variables. Table 2 shows examples of non-causal (e.g., diagnostic and prognostic) and causal (e.g., intervention and etiologic) clinical studies. Concordance between the research question, its aim, and the choice of theoretical design will provide a strong foundation and the right direction for the research process and path. 

**Table 2:** Examples of clinical study titles according to the category of research and the data collection designs

Research Category	Study Title
Diagnostic	Plasma Concentration of B-type Natriuretic Peptide (BNP) in the Diagnosis of Left Ventricular Dysfunction
	The Centor and McIsaac Scores and the Group A Streptococcal Pharyngitis
Prognostic	The Apgar Score and Infant Mortality
	SCORE (Systematic COronary Risk Evaluation) for the Estimation of Ten-Year Risk of Fatal Cardiovascular Disease
Intervention	Dexamethasone in Very Low Birth Weight Infants
	Bariatric Surgery of Obesity in Type 2 Diabetes and Metabolic Syndrome
Etiologic	Thalidomide and Reduction Deformities of the Limbs
	Work Stress and Risk of Cardiovascular Mortality

A problem in clinical epidemiology is phrased in a mathematical relationship below, where the outcome is a function of the determinant (D) conditional on the extraneous determinants (ED) or more commonly known as the confounding factors [7]:

For non-causal research, Outcome = f (D1, D2…Dn) For causal research, Outcome = f (D | ED)

A fine research question is composed of at least three components: 1) an outcome or a health condition, 2) determinant/s or associated factors to the outcome, and 3) the domain. The outcome and the determinants have to be clearly conceptualized and operationalized as measurable variables (Table 3; PICOT [14] and FINER [15]). The study domain is the theoretical source population from which the study population will be sampled, similar to the wording on a drug package insert that reads, “use this medication (study results) in people with this disease” [7].

**Table 3:** The PICOT and FINER of a research question

Acronym	Explanation
P =	Patient (or the domain)
I =	Intervention or treatment (or the determinants in non-experimental)
C =	Comparison (only in experimental)
O =	Outcome
T =	Time describes the duration of data collection
F =	Feasible with the current and/or potential available resources
I =	Important and interesting to current clinical practice and to you, respectively
N =	Novel and adding to the existing corpus of scientific knowledge
E =	Ethical research conducted without harm to participants and institutions
R =	Relevant to as many parties as possible, not only to your own practice

The interpretation of study results as they apply to wider populations is known as generalization, and generalization can either be statistical or made using scientific inferences [16]. Generalization supported by statistical inferences is seen in studies on disease prevalence where the sample population is representative of the source population. By contrast, generalizations made using scientific inferences are not bound by the representativeness of the sample in the study; rather, the generalization should be plausible from the underlying scientific mechanisms as long as the study design is valid and nonbiased. Scientific inferences and generalizations are usually the aims of causal studies. 

**Figure 2:**
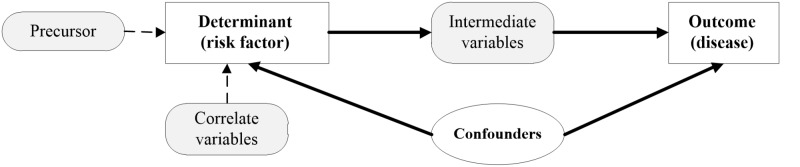
The confounders in a causal relationship

Confounding: Confounding is a situation where true effects are obscured or confused [7,16]. Confounding variables or confounders affect the validity of a study’s outcomes and should be prevented or mitigated in the planning stages and further managed in the analytical stages. Confounders are also known as extraneous determinants in epidemiology due to their inherent and simultaneous relationships to both the determinant and outcome (Figure 2), which are usually one-determinant-to-one outcome in causal clinical studies. The known confounders are also called observed confounders. These can be minimized using randomization, restriction, or a matching strategy. Residual confounding has occurred in a causal relationship when identified confounders were not measured accurately. Unobserved confounding occurs when the confounding effect is present as a variable or factor not observed or yet defined and, thus, not measured in the study. Age and gender are almost universal confounders followed by ethnicity and socio-economic status.

Confounders have three main characteristics. They are a potential risk factor for the disease, associated with the determinant of interest, and should not be an intermediate variable between the determinant and the outcome or a precursor to the determinant. For example, a sedentary lifestyle is a cause for acute coronary syndrome (ACS), and smoking could be a confounder but not cardiorespiratory unfitness (which is an intermediate factor between a sedentary lifestyle and ACS). For patients with ACS, not having a pair of sports shoes is not a confounder – it is a correlate for the sedentary lifestyle. Similarly, depression would be a precursor, not a confounder.

Sample size consideration: Sample size calculation provides the required number of participants to be recruited in a new study to detect true differences in the target population if they exist. Sample size calculation is based on three facets: an estimated difference in group sizes, the probability of α (Type I) and β (Type II) errors chosen based on the nature of the treatment or intervention, and the estimated variability (interval data) or proportion of the outcome (nominal data) [17-18]. The clinically important effect sizes are determined based on expert consensus or patients’ perception of benefit. Value and economic consideration have increasingly been included in sample size estimations. Sample size and the degree to which the sample represents the target population affect the accuracy and generalization of a study’s reported effects. 

Pilot study: Pilot studies assess the feasibility of the proposed research procedures on small sample size. Pilot studies test the efficiency of participant recruitment with minimal practice or service interruptions. Pilot studies should not be conducted to obtain a projected effect size for a larger study population because, in a typical pilot study, the sample size is small, leading to a large standard error of that effect size. This leads to bias when projected for a large population. In the case of underestimation, this could lead to inappropriately terminating the full-scale study. As the small pilot study is equally prone to bias of overestimation of the effect size, this would lead to an underpowered study and a failed full-scale study [19]. 

The Design of Data Collection

The “perfect” study design in the theoretical phase now faces the practical and realistic challenges of feasibility. This is the step where different methods for data collection are considered, with one selected as the most appropriate based on the theoretical design along with feasibility and efficiency. The goal of this stage is to achieve the highest possible validity with the lowest risk of biases given available resources and existing constraints. 

In causal research, data on the outcome and determinants are collected with utmost accuracy via a strict protocol to maximize validity and precision. The validity of an instrument is defined as the degree of fidelity of the instrument, measuring what it is intended to measure, that is, the results of the measurement correlate with the true state of an occurrence. Another widely used word for validity is accuracy. Internal validity refers to the degree of accuracy of a study’s results to its own study sample. Internal validity is influenced by the study designs, whereas the external validity refers to the applicability of a study’s result in other populations. External validity is also known as generalizability and expresses the validity of assuming the similarity and comparability between the study population and the other populations. Reliability of an instrument denotes the extent of agreeableness of the results of repeated measurements of an occurrence by that instrument at a different time, by different investigators or in a different setting. Other terms that are used for reliability include reproducibility and precision. Preventing confounders by identifying and including them in data collection will allow statistical adjustment in the later analyses. In descriptive research, outcomes must be confirmed with a referent standard, and the determinants should be as valid as those found in real clinical practice.

Common designs for data collection include cross-sectional, case-control, cohort, and randomized controlled trials (RCTs). Many other modern epidemiology study designs are based on these classical study designs such as nested case-control, case-crossover, case-control without control, and stepwise wedge clustered RCTs. A cross-sectional study is typically a snapshot of the study population, and an RCT is almost always a prospective study. Case-control and cohort studies can be retrospective or prospective in data collection. The nested case-control design differs from the traditional case-control design in that it is “nested” in a well-defined cohort from which information on the cohorts can be obtained. This design also satisfies the assumption that cases and controls represent random samples of the same study base. Table 4 provides examples of these data collection designs.

**Table 4:** Examples of clinical study titles according to the data collection designs

Data Collection Designs	Study Title
Cross-sectional	The National Health and Morbidity Survey (NHMS)
	The National Health and Nutrition Examination Survey (NHANES)
Cohort	Framingham Heart Study
	The Malaysian Cohort (TMC) project
Case-control	A Case-Control Study of the Effectiveness of Bicycle Safety Helmets
	Open-Angle Glaucoma and Ocular Hypertension: the Long Island Glaucoma Case-Control Study
Nested case-control	Nurses' Health Study on Plasma Adipokines and Endometriosis Risk
	Physicians' Health Study Plasma Homocysteine and Risk of Myocardial Infarction
Randomized controlled trial	The Women’s Health Initiative
	U.K. Prospective Diabetes Study
Cross-over	Intranasal-agonist in Allergic Rhinitis Published in the Allergy in 2000
	Effect of Palm-based Tocotrienols and Tocopherol Mixture Supplementation on Platelet Aggregation in Subjects with Metabolic Syndrome

Additional aspects in data collection: No single design of data collection for any research question as stated in the theoretical design will be perfect in actual conduct. This is because of myriad issues facing the investigators such as the dynamic clinical practices, constraints of time and budget, the urgency for an answer to the research question, and the ethical integrity of the proposed experiment. Therefore, feasibility and efficiency without sacrificing validity and precision are important considerations in data collection design. Therefore, data collection design requires additional consideration in the following three aspects: experimental/non-experimental, sampling, and timing [7]:

Experimental or non-experimental: Non-experimental research (i.e., “observational”), in contrast to experimental, involves data collection of the study participants in their natural or real-world environments. Non-experimental researches are usually the diagnostic and prognostic studies with cross-sectional in data collection. The pinnacle of non-experimental research is the comparative effectiveness study, which is grouped with other non-experimental study designs such as cross-sectional, case-control, and cohort studies [20]. It is also known as the benchmarking-controlled trials because of the element of peer comparison (using comparable groups) in interpreting the outcome effects [20]. Experimental study designs are characterized by an intervention on a selected group of the study population in a controlled environment, and often in the presence of a similar group of the study population to act as a comparison group who receive no intervention (i.e., the control group). Thus, the widely known RCT is classified as an experimental design in data collection. An experimental study design without randomization is referred to as a quasi-experimental study. Experimental studies try to determine the efficacy of a new intervention on a specified population. Table 5 presents the advantages and disadvantages of experimental and non-experimental studies [21].

**Table 5:** The advantages and disadvantages of experimental and non-experimental data collection designs ^a^May be an issue in cross-sectional studies that require a long recall to the past such as dietary patterns, antenatal events, and life experiences during childhood.

Non-experimental	Experimental
Advantages	
Quick results are possible	Comparable groups
Relatively less costly	Hawthorne and placebo effects mitigated
No recall bias^a^	Straightforward, robust statistical analysis
No time effects	Convincing results as evidence
Real-life data	
Disadvantages	
Observed, unobserved, and residual confounding	Expensive
	Time-consuming
	Overly controlled environment
	Loss to follow-up
	Random allocation of potentially harmful treatment may not be ethically permissible

Once an intervention yields a proven effect in an experimental study, non-experimental and quasi-experimental studies can be used to determine the intervention’s effect in a wider population and within real-world settings and clinical practices. Pragmatic or comparative effectiveness are the usual designs used for data collection in these situations [22].

Sampling/census: Census is a data collection on the whole source population (i.e., the study population is the source population). This is possible when the defined population is restricted to a given geographical area. A cohort study uses the census method in data collection. An ecologic study is a cohort study that collects summary measures of the study population instead of individual patient data. However, many studies sample from the source population and infer the results of the study to the source population for feasibility and efficiency because adequate sampling provides similar results to the census of the whole population. Important aspects of sampling in research planning are sample size and representation of the population. Sample size calculation accounts for the number of participants needed to be in the study to discover the actual association between the determinant and outcome. Sample size calculation relies on the primary objective or outcome of interest and is informed by the estimated possible differences or effect size from previous similar studies. Therefore, the sample size is a scientific estimation for the design of the planned study.

A sampling of participants or cases in a study can represent the study population and the larger population of patients in that disease space, but only in prevalence, diagnostic, and prognostic studies. Etiologic and interventional studies do not share this same level of representation. A cross-sectional study design is common for determining disease prevalence in the population. Cross-sectional studies can also determine the referent ranges of variables in the population and measure change over time (e.g., repeated cross-sectional studies). Besides being cost- and time-efficient, cross-sectional studies have no loss to follow-up; recall bias; learning effect on the participant; or variability over time in equipment, measurement, and technician. A cross-sectional design for an etiologic study is possible when the determinants do not change with time (e.g., gender, ethnicity, genetic traits, and blood groups). 

In etiologic research, comparability between the exposed and the non-exposed groups is more important than sample representation. Comparability between these two groups will provide an accurate estimate of the effect of the exposure (risk factor) on the outcome (disease) and enable valid inference of the causal relation to the domain (the theoretical population). In a case-control study, a sampling of the control group should be taken from the same study population (study base), have similar profiles to the cases (matching) but do not have the outcome seen in the cases. Matching important factors minimizes the confounding of the factors and increases statistical efficiency by ensuring similar numbers of cases and controls in confounders’ strata [23-24]. Nonetheless, perfect matching is neither necessary nor achievable in a case-control study because a partial match could achieve most of the benefits of the perfect match regarding a more precise estimate of odds ratio than statistical control of confounding in unmatched designs [25-26]. Moreover, perfect or full matching can lead to an underestimation of the point estimates [27-28].

**Figure 3:**
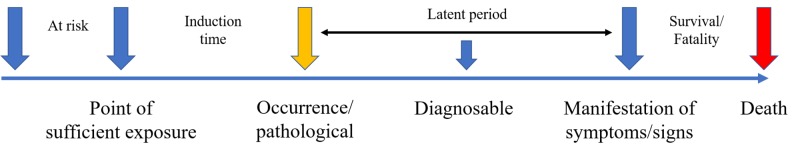
The time features of a variable

Time feature: The timing of data collection for the determinant and outcome characterizes the types of studies. A cross-sectional study has the axis of time zero (T = 0) for both the determinant and the outcome, which separates it from all other types of research that have time for the outcome T > 0. Retrospective or prospective studies refer to the direction of data collection. In retrospective studies, information on the determinant and outcome have been collected or recorded before. In prospective studies, this information will be collected in the future. These terms should not be used to describe the relationship between the determinant and the outcome in etiologic studies. Time of exposure to the determinant, the time of induction, and the time at risk for the outcome are important aspects to understand. Time at risk is the period of time exposed to the determinant risk factors. Time of induction is the time from the sufficient exposure to the risk or causal factors to the occurrence of a disease. The latent period is when the occurrence of a disease without manifestation of the disease such as in “silence” diseases for example cancers, hypertension and type 2 diabetes mellitus which is detected from screening practices. Figure 3 illustrates the time features of a variable. Variable timing is important for accurate data capture. 

The Design of Statistical Analysis

Statistical analysis of epidemiologic data provides the estimate of effects after correcting for biases (e.g., confounding factors) measures the variability in the data from random errors or chance [7,16,29]. An effect estimate gives the size of an association between the studied variables or the level of effectiveness of an intervention. This quantitative result allows for comparison and assessment of the usefulness and significance of the association or the intervention between studies. This significance must be interpreted with a statistical model and an appropriate study design. Random errors could arise in the study resulting from unexplained personal choices by the participants. Random error is, therefore, when values or units of measurement between variables change in non-concerted or non-directional manner. Conversely, when these values or units of measurement between variables change in a concerted or directional manner, we note a significant relationship as shown by statistical significance. 

Variability: Researchers almost always collect the needed data through a sampling of subjects/participants from a population instead of a census. The process of sampling or multiple sampling in different geographical regions or over different periods contributes to varied information due to the random inclusion of different participants and chance occurrence. This sampling variation becomes the focus of statistics when communicating the degree and intensity of variation in the sampled data and the level of inference in the population. Sampling variation can be influenced profoundly by the total number of participants and the width of differences of the measured variable (standard deviation). Hence, the characteristics of the participants, measurements and sample size are all important factors in planning a study.

Statistical strategy: Statistical strategy is usually determined based on the theoretical and data collection designs. Use of a prespecified statistical strategy (including the decision to dichotomize any continuous data at certain cut-points, sub-group analysis or sensitive analyses) is recommended in the study proposal (i.e., protocol) to prevent data dredging and data-driven reports that predispose to bias. The nature of the study hypothesis also dictates whether directional (one-tailed) or non-directional (two-tailed) significance tests are conducted. In most studies, two-sided tests are used except in specific instances when unidirectional hypotheses may be appropriate (e.g., in superiority or non-inferiority trials). While data exploration is discouraged, epidemiological research is, by nature of its objectives, statistical research. Hence, it is acceptable to report the presence of persistent associations between any variables with plausible underlying mechanisms during the exploration of the data. The statistical methods used to produce the results should be explicitly explained. Many different statistical tests are used to handle various kinds of data appropriately (e.g., interval vs discrete), and/or the various distribution of the data (e.g., normally distributed or skewed). For additional details on statistical explanations and underlying concepts of statistical tests, readers are recommended the references as cited in this sentence [30-31]. 

**Figure 4:**
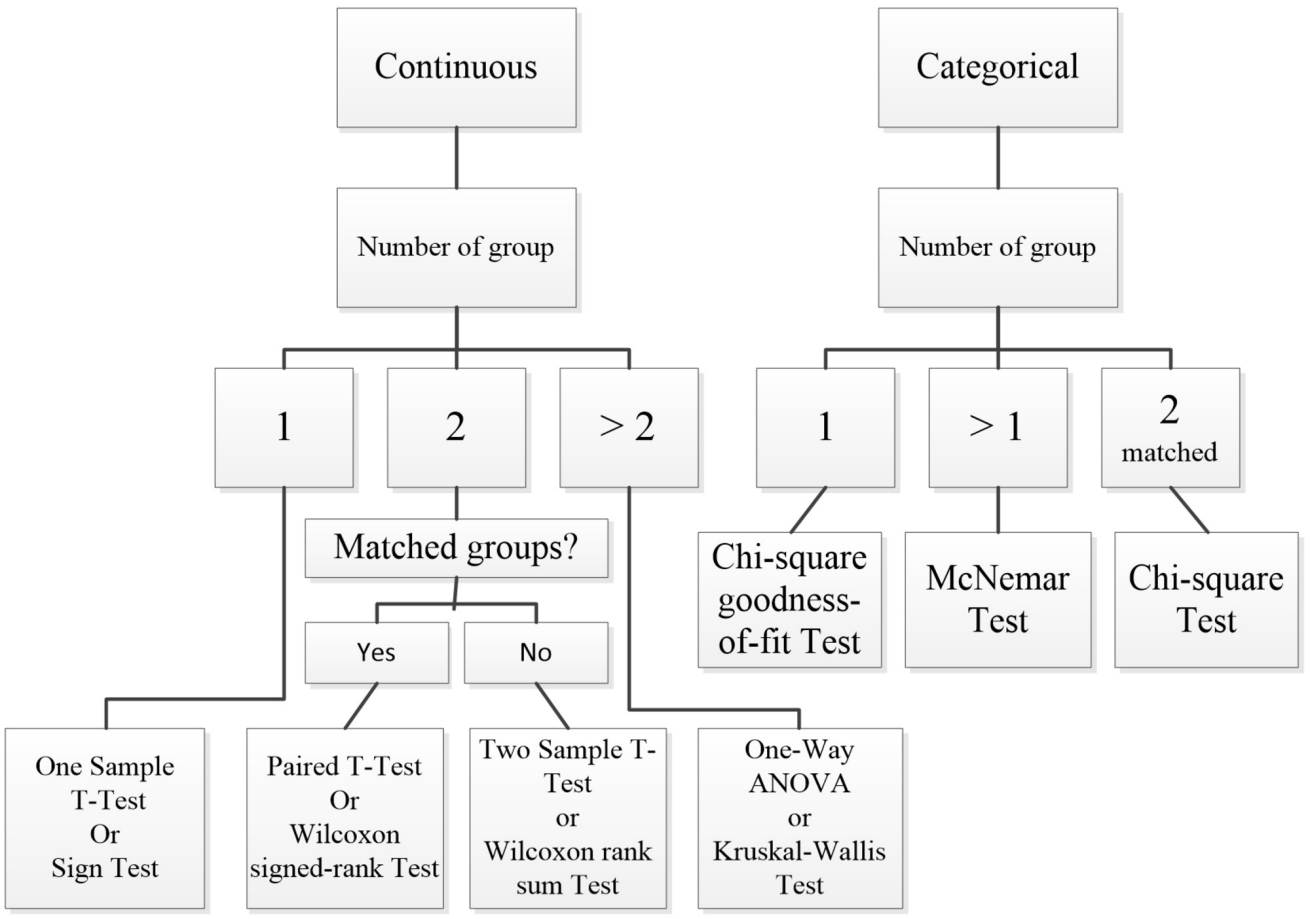
Statistical tests available for analyses of different types of data

Steps in statistical analyses: Statistical analysis begins with checking for data entry errors. Duplicates are eliminated, and proper units should be confirmed. Extremely low, high or suspicious values are confirmed from the source data again. If this is not possible, this is better classified as a missing value. However, if the unverified suspicious data are not obviously wrong, they should be further examined as an outlier in the analysis. The data checking and cleaning enables the analyst to establish a connection with the raw data and to anticipate possible results from further analyses. This initial step involves descriptive statistics that analyze central tendency (i.e., mode, median, and mean) and dispersion (i.e., (minimum, maximum, range, quartiles, absolute deviation, variance, and standard deviation) of the data. Certain graphical plotting such as scatter plot, a box-whiskers plot, histogram or normal Q-Q plot are helpful at this stage to verify data normality in distribution. See Figure 4 for the statistical tests available for analyses of different types of data.

Once data characteristics are ascertained, further statistical tests are selected. The analytical strategy sometimes involves the transformation of the data distribution for the selected tests (e.g., log, natural log, exponential, quadratic) or for checking the robustness of the association between the determinants and their outcomes. This step is also referred to as inferential statistics whereby the results are about hypothesis testing and generalization to the wider population that the study’s sampled participants represent. The last statistical step is checking whether the statistical analyses fulfill the assumptions of that particular statistical test and model to avoid violation and misleading results. These assumptions include evaluating normality, variance homogeneity, and residuals included in the final statistical model. Other statistical values such as Akaike information criterion, variance inflation factor/tolerance, and R2 are also considered when choosing the best-fitted models. Transforming raw data could be done, or a higher level of statistical analyses can be used (e.g., generalized linear models and mixed-effect modeling). Successful statistical analysis allows conclusions of the study to fit the data. 

Bayesian and Frequentist statistical frameworks: Most of the current clinical research reporting is based on the frequentist approach and hypotheses testing p values and confidence intervals. The frequentist approach assumes the acquired data are random, attained by random sampling, through randomized experiments or influences, and with random errors. The distribution of the data (its point estimate and confident interval) infers a true parameter in the real population. The major conceptual difference between Bayesian statistics and frequentist statistics is that in Bayesian statistics, the parameter (i.e., the studied variable in the population) is random and the data acquired is real (true or fix). Therefore, the Bayesian approach provides a probability interval for the parameter. The studied parameter is random because it could vary and be affected by prior beliefs, experience or evidence of plausibility. In the Bayesian statistical approach, this prior belief or available knowledge is quantified into a probability distribution and incorporated into the acquired data to get the results (i.e., the posterior distribution). This uses mathematical theory of Bayes’ Theorem to “turn around” conditional probabilities.

Reporting

The goal of research reporting is to present findings succinctly and timely via conference proceedings or journal publication. Concise and explicit language use, with all the necessary details to enable replication and judgment of the study applicability, are the guiding principles in clinical studies reporting.

Writing for Reporting

Medical writing is very much a technical chore that accommodates little artistic expression. Research reporting in medicine and health sciences emphasize clear and standardized reporting, eschewing adjectives and adverbs extensively used in popular literature. Regularly reviewing published journal articles can familiarize authors with proper reporting styles and help enhance writing skills. Authors should familiarize themselves with standard, concise, and appropriate rhetoric for the intended audience, which includes consideration for journal reviewers, editors, and referees. However, proper language can be somewhat subjective. While each publication may have varying requirements for submission, the technical requirements for formatting an article are usually available via author or submission guidelines provided by the target journal. 

Research reports for publication often contain a title, abstract, introduction, methods, results, discussion, and conclusions section, and authors may want to write each section in sequence. However, best practices indicate the abstract and title should be written last. Authors may find that when writing one section of the report, ideas come to mind that pertains to other sections, so careful note taking is encouraged. One effective approach is to organize and write the result section first, followed by the discussion and conclusions sections. Once these are drafted, write the introduction, abstract, and the title of the report. Regardless of the sequence of writing, the author should begin with a clear and relevant research question to guide the statistical analyses, result interpretation, and discussion. The study findings can be a motivator to propel the author through the writing process, and the conclusions can help the author draft a focused introduction.

Writing for Publication

Specific recommendations on effective medical writing and table generation are available [32]. One such resource is Effective Medical Writing: The Write Way to Get Published, which is an updated collection of medical writing articles previously published in the Singapore Medical Journal [33]. The British Medical Journal’s Statistics Notes series also elucidates common and important statistical concepts and usages in clinical studies. Writing guides are also available from individual professional societies, journals, or publishers such as Chest (American College of Physicians) medical writing tips, PLoS Reporting guidelines collection, Springer’s Journal Author Academy, and SAGE’s Research methods [34-37]. Standardized research reporting guidelines often come in the form of checklists and flow diagrams. Table 6 presents a list of reporting guidelines. A full compilation of these guidelines is available at the EQUATOR (Enhancing the QUAlity and Transparency Of health Research) Network website [38] which aims to improve the reliability and value of medical literature by promoting transparent and accurate reporting of research studies. Publication of the trial protocol in a publicly available database is almost compulsory for publication of the full report in many potential journals.

**Table 6:** Examples of reporting guidelines and checklists

No.	Reporting Guidelines and Checklists	
	CONSORT - CONsolidated Standards Of Reporting Trials	
	A 25-item checklist for reporting of randomized controlled trials. There are appropriate extensions to the CONSORT statement due to variations in the standard trial methodology such as different design aspects (e.g., cluster, pragmatic, non-inferiority and equivalence trials), interventions (e.g., herbals) and data (e.g., harms, including the extension for writing abstracts)	
	SPIRIT - Standard Protocol Items: Recommendations for Interventional Trials	
	A 33-item checklist for reporting protocols for randomized controlled trials	
	COREQ - COnsolidated criteria for REporting Qualitative research	
	A 32-item checklist for reporting qualitative research of interviews and focus groups	
	STARD - STAndards for the Reporting of Diagnostic accuracy studies	
	A 25-item checklist for reporting of diagnostic accuracy studies	
	PRISMA - Preferred Reporting Items for Systematic reviews and Meta-Analyses	
	A 27-item checklist for reporting of systematic reviews	
	PRISMA-P - Preferred Reporting Items for Systematic reviews and Meta-Analyses Protocols	
	A 17-item checklist for reporting of systematic review and meta-analysis protocols	
	MOOSE - Meta-analysis Of Observational Studies in Epidemiology	
	A 35-item checklist for reporting of meta-analyses of observational studies	
	STROBE - STrengthening the Reporting of OBservational studies in Epidemiology	
	For reporting of observational studies in epidemiology	
		Checklist for cohort, case-control and cross-sectional studies (combined)
		Checklist for cohort studies
		Checklist for case-control studies
		Checklist for cross-sectional studies
	Extensions of the STROBE statement	
	STROME-ID - STrengthening the Reporting Of Molecular Epidemiology for Infectious Diseases	
	A 42-item checklist	
	STREGA - STrengthening the REporting of Genetic Associations	
	A 22-item checklist for reporting of gene-disease association studies	
	CHEERS - Consolidated Health Economic Evaluation Reporting Standards	
	A 24-item checklist for reporting of health economic evaluations	

Graphics and Tables

Graphics and tables should emphasize salient features of the underlying data and should coherently summarize large quantities of information. Although graphics provide a break from dense prose, authors must not forget that these illustrations should be scientifically informative, not decorative. The titles for graphics and tables should be clear, informative, provide the sample size, and use minimal font weight and formatting only to distinguish headings, data entry or to highlight certain results. Provide a consistent number of decimal points for the numerical results, and with no more than four for the P value. Most journals prefer cell-delineated tables created using the table function in word processing or spreadsheet programs. Some journals require specific table formatting such as the absence or presence of intermediate horizontal lines between cells.

Authorship

Decisions of authorship are both sensitive and important and should be made at an early stage by the study’s stakeholders. Guidelines and journals’ instructions to authors abound with authorship qualifications. The guideline on authorship by the International Committee of Medical Journal Editors is widely known and provides a standard used by many medical and clinical journals [39]. Generally, authors are those who have made major contributions to the design, conduct, and analysis of the study, and who provided critical readings of the manuscript (if not involved directly in manuscript writing). 

Picking a target journal for submission

Once a report has been written and revised, the authors should select a relevant target journal for submission. Authors should avoid predatory journals—publications that do not aim to advance science and disseminate quality research. These journals focus on commercial gain in medical and clinical publishing. Two good resources for authors during journal selection are Think-Check-Submit and the defunct Beall's List of Predatory Publishers and Journals (now archived and maintained by an anonymous third-party) [40,41]. Alternatively, reputable journal indexes such as Thomson Reuters Journal Citation Reports, SCOPUS, MedLine, PubMed, EMBASE, EBSCO Publishing's Electronic Databases are available areas to start the search for an appropriate target journal. Authors should review the journals’ names, aims/scope, and recently published articles to determine the kind of research each journal accepts for publication. Open-access journals almost always charge article publication fees, while subscription-based journals tend to publish without author fees and instead rely on subscription or access fees for the full text of published articles.

## Conclusions

Conducting a valid clinical research requires consideration of theoretical study design, data collection design, and statistical analysis design. Proper study design implementation and quality control during data collection ensures high-quality data analysis and can mitigate bias and confounders during statistical analysis and data interpretation. Clear, effective study reporting facilitates dissemination, appreciation, and adoption, and allows the researchers to affect real-world change in clinical practices and care models. Neutral or absence of findings in a clinical study are as important as positive or negative findings. Valid studies, even when they report an absence of expected results, still inform scientific communities of the nature of a certain treatment or intervention, and this contributes to future research, systematic reviews, and meta-analyses. Reporting a study adequately and comprehensively is important for accuracy, transparency, and reproducibility of the scientific work as well as informing readers.

## References

[1] Ioannidis JPA (2005). Why most published research findings are false. PLoS Med.

[2] Ioannidis JPA (2014). How to make more published research true. PLoS Med.

[3] Chalmers I, Glasziou P (2009). Avoidable waste in the production and reporting of research evidence. Lancet.

[4] Charatan F The truth about the drug companies: how they deceive us and what to do about it. BMJ.

[5] Altman DG (1994). The scandal of poor medical research. BMJ.

[6] Smith R (1995). Their lordships on medical research. BMJ.

[7] Grobbee DE, Hoes AW (2014). Clinical Epidemiology: Principles, Methods, and Applications for Clinical Research.

[8] Chalmers I, Bracken MB, Djulbegovic B (2014). How to increase value and reduce waste when research priorities are set. Lancet.

[9] Clarke M (2004). Doing new research? Don't forget the old. PLoS Med.

[10] Roberts I, Ker K (2015). How systematic reviews cause research waste. Lancet.

[11] Bordage G (2009). Conceptual frameworks to illuminate and magnify. Med Educ.

[12] O'Brien BC, Ruddick VJ, Young JQ (2016). Generating research questions appropriate for qualitative studies in health professions education. Acad Med.

[13] Greenhalgh T (2014). How to Read a Paper: The Basics of Evidence-Based Medicine (How - How To). https://books.google.com/books?id=q-92iFMFJGwC&lpg=PT10&ots=AigQt3qdT_&dq=How%20to%20Read%20a%20Paper%3A%20The%20Basics%20of%20Evidence-Based%20Medicine%20(How%20-%20How%20To)&lr&pg=PT10#v=onepage&q=How%20to%20Read%20a%20Paper:%20The%20Basics%20of%20Evidence-Based%20Medicine%20(How%20-%20How%20To)&f=false.

[14] Guyatt G, Drummond R, Meade M, Cook D (2008). The Evidence Based-Medicine Working Group Users’ Guides to the Medical Literature.

[15] Dine CJ, Shea JA, Kogan JR (2016). Generating good research questions in health professions education. Acad Med.

[16] Rothman KJ (2012). Epidemiology: An Introduction.

[17] Florey CD (1993). Sample size for beginners. BMJ.

[18] Campbell MJ, Julious SA, Altman DG (1995). Estimating sample sizes for binary, ordered categorical, and continuous outcomes in two group comparisons. BMJ.

[19] Kraemer HC, Mintz J, Noda A, Tinklenberg J, Yesavage JA (2006). Caution regarding the use of pilot studies to guide power calculations for study proposals. Arch Gen Psychiatry.

[20] Malmivaara A (2015). Benchmarking controlled trial-a novel concept covering all observational effectiveness studies. Ann Med.

[21] Glasgow RE, Vogt TM, Boles SM (1999). Evaluating the public health impact of health promotion interventions: the RE-AIM framework. Am J Public Health.

[22] Patsopoulos NA (2011). A pragmatic view on pragmatic trials. Dialogues Clinical Neurosci.

[23] Rose S, Laan MJ (2009). Why match? Investigating matched case-control study designs with causal effect estimation. Int J Biostat.

[24] Pearce N (2016). Analysis of matched case-control studies. BMJ.

[25] Sturmer T, Brenner H (2001). Degree of matching and gain in power and efficiency in case-control studies. Epidemiol.

[26] Friedlander Y, Merom DL, Kark JD (1993). A comparison of different matching designs in case-control studies: an empirical example using continuous exposures, continuous confounders and incidence of myocardial infarction. Stat Med.

[27] de Graaf MA, Jager KJ, Zoccali C, Dekker FW (2011). Matching, an appealing method to avoid confounding?. Nephron Clin Pract.

[28] Costanza MC (1995). Matching. Prev Med.

[29] Haynes RB, Sackett DL, Guyatt GH, Tugwell P (2006). Clinical Epidemiology: How to Do Clinical Practice Research.

[30] Petrie A (1988). Lecture Notes on Medical Statistics.

[31] Kirkwood B, Sterne J (2003). Essential Medical Statistics (Essentials).

[32] Hall GM (2012). How to Write a Paper.

[33] Peh WCG, Ng KH (2010). Effective Medical Writing: The Write Way To Get Published (UM Press).

[34] (2019). Medical writing tip: CHEST journal. http://journal.publications.chestnet.org/collection.aspx.

[35] PLOS Collections (2019). Article collections published by the Public Library of Science. http://www.ploscollections.org/article/browse/issue/info.

[36] (2019). Journal author academy: Springer. http://www.springer.com/gp/authors-editors/journal-author/journal-author-academy.

[37] (2019). SAGE research methods. http://srmo.sagepub.com/.

[38] (2019). Enhancing the quality and transparency of health research. http://www.equator-network.org/.

[39] (2019). International Committee of Medical Journal Editors. http://www.icmje.org/.

[40] (2019). Think check submit. https://thinkchecksubmit.org/.

[41] (2019). (Archived) Beall's list of predatory journals and publishers. https://beallslist.weebly.com/.

